# Development and Validation of a New Eco-Friendly HPLC-PDA Bioanalytical Method for Studying Pharmacokinetics of Seliciclib

**DOI:** 10.3390/medicina60101686

**Published:** 2024-10-14

**Authors:** Reem M. Abuhejail, Nourah Z. Alzoman, Ibrahim A. Darwish

**Affiliations:** Department of Pharmaceutical Chemistry, College of Pharmacy, King Saud University, P.O. Box 2457, Riyadh 11451, Saudi Arabia

**Keywords:** seliciclib, cyclin-dependent kinase inhibitors, HPLC-PDA, pharmacokinetic study, therapeutic drug monitoring, green analytical chemistry

## Abstract

*Background and Objectives:* Seliciclib (SEL) is the first selective, orally bioavailable potential drug containing cyclin-dependent kinase inhibitors. Preclinical studies showed antitumor activity in a broad range of human tumor xenografts, neurodegenerative diseases, renal dysfunctions, viral infections, and chronic inflammatory disorders. To support the pharmacokinetics and aid in therapeutic monitoring of SEL following its administration for therapy, an efficient analytical tool capable of quantifying the concentrations of SEL in blood plasma is needed. In the literature, there is no existing method for quantifying SEL in plasma samples. This study introduces the first HPLC method with a photodiode array (PDA) detector for the quantitation of SEL in plasma. *Materials and Methods:* The chromatographic resolution of SEL and linifanib as an internal standard (IS) was achieved on Zorbax Eclipse Plus C18 HPLC column (150 mm length × 4.6 mm internal diameter, 5 µm particle size), with a mobile phase composed of acetonitrile–ammonium acetate, pH 5 (50:50, *v*/*v*) at a flow rate of 1.0 mL min^−1^. Both SEL and IS were detected by PDA at 230 nm. The method was validated according to the ICH guidelines for bioanalytical method validation. *Results:* The method exhibited linearity in concentrations ranging from 50 to 1000 ng mL^−1^, with a limit of quantitation of 66.1 ng mL^−1^. All remaining validation parameters satisfied the ICH validation criteria. The environmental sustainability of the method was verified using three extensive tools. The proposed HPLC-PDA method was effectively utilized to study the pharmacokinetics of SEL in rats after a single oral administration of 25 mg/kg. *Conclusions:* The proposed method stands as a valuable tool for studying SELs for pharmacokinetics in humans. It aids in achieving the targeted therapeutic advantages and safety of treatment with SEL by optimizing the SEL dosage and dosing schedule.

## 1. Introduction

**Figure 1:**
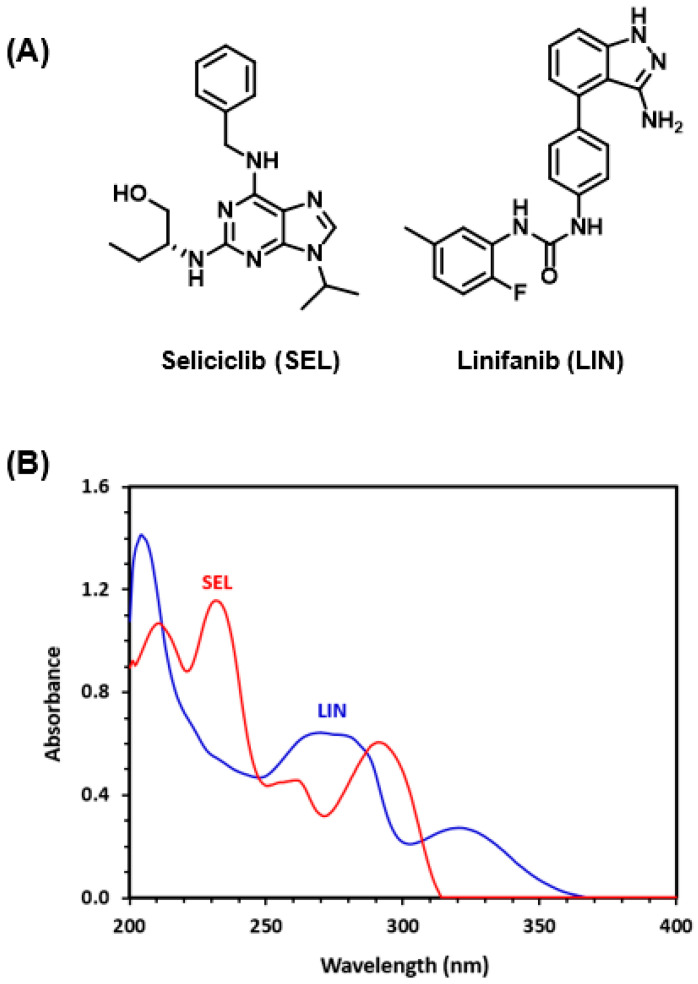
The chemical structures and abbreviations of seliciclib and linifanib (**A**), and the UV absorption spectra of their methanolic solutions (**B**). The concentrations of these solutions were 20 and 10 µg mL^−1^ for SEL and LIN, respectively.

Seleciclib (SLC) is a purine analog small molecule (Figure 1) with the chemical name (2R)-2-{[6-(Benzylamino)-9-(propan-2-yl)-9H-purin-2-yl]amino}butan-1-ol. It has a chemical formula of C_19_H_26_N_6_O and a molar mass of 354.46 g/mole [1]. SEL was developed by Cyclacel Pharmaceutical Inc. (New Jersey, NJ, USA) for the potential treatment of different cancer types like non-small cell lung cancer, leukemia, nasopharyngeal cancer, and lymphoma. It has also shown promise in managing neurodegenerative diseases, renal dysfunctions, various viral infections such as human immunodeficiency virus and herpes simplex, as well as chronic inflammatory conditions [2]. SLC belongs to the family of cyclin-dependent kinase (CDK) inhibitor drugs. CDKs are proteins that regulate the cell cycle by acting as serine/threonine kinases. SLC competitively binds to CDK 2, 7, and 9 at their ATP binding sites, inhibiting their function and halting cell cycle progression. Furthermore, SLC disrupts the CDK-mediated phosphorylation of the carboxy-terminal domain of RNA polymerase II, leading to the inhibition of RNA polymerase II-dependent transcription. This inhibition lowers the levels of anti-apoptotic effects, such as myeloid cell leukemia sequence 1 (Mcl-1), that are crucial for the survival of various tumor cell types. The reduction in anti-apoptotic factors triggers apoptosis, enhancing the antiproliferative effects of SLC [3,4].

The promising clinical outcomes and therapeutic advantages of SLC raise hopes of regulatory approval from concerned authorities like the Food and Drug Administration (FDA), enabling pharmaceutical companies to introduce new SLC formulations to the market. Indeed, the effectiveness and safety of treatment with SEL rely on the quality of its formulation and the assessment of its plasma levels in patients throughout therapy. Many analytical methods were developed for the characterization and quantitation of SEL in its bulk drug substance and/or formulations [5,6,7]; however, there is no available validated method for quantifying SEL in plasma, enabling studying its pharmacokinetics. Therefore, there is an immediate requirement for the development and validation of a new method to quantify SEL concentrations in human plasma samples with simple sample pretreatment procedures, adequate sensitivity, and high accuracy. High-performance liquid chromatography (HPLC) with a photodiode array detector (PDA) offers several advantages in clinical analysis. One key benefit is its ability to provide spectral information, allowing for the specific detection of analytes in a complex matrix (e.g., plasma) based on their light absorption spectra. Additionally, the PDA offers high sensitivity, enabling the quantitation of analytes at low concentrations. This high sensitivity, coupled with the separation capabilities of HPLC, makes HPLC-PDA a valuable tool in clinical settings for the accurate quantification of drugs in biological samples.

This study describes the development and validation of the first HPLC-PDA method for the quantitation of SEL in plasma samples. The method has high sensitivity, enabling the quantitation of SEL at the nanogram level (the limit of quantitation is 66.1 ng mL^−1^). The method involved a remarkably simple and swift non-extractive preparation of plasma samples using protein precipitation with methanol. The method was fully validated and successfully utilized for the accurate quantification of SEL in human plasma samples and for assessing the pharmacokinetics of SEL in rats.

## 2. Materials and Methods

### 2.1. Instruments

We used the HPLC system manufactured by Shimadzu Corporation (Kyoto, Japan), equipped with an LC-10AD VP pump alongside an FCV-10AL VP low-pressure flow-control valve, an SCL-10A VP system controller, an SIL-30AC autosampler, and an LC-20AD photodiode array detector set to 230 nm. Control and data acquisition were performed utilizing Shimadzu Corporation’s LCsolution software (version 1.25), which was provided with the HPLC system. The pH of buffer solutions was calibrated using a pH meter (model pH 211 by Hanna, Nusfalau, Romania). Buffer solutions’ pH was adjusted using a pH meter (pH 211: Hanna, Nusfalau, Romania). Standards were mixed with plasma samples using a Heidolph vortex mixer (D-91126; Schwabach, Germany), while a Sigma centrifuge (1–15PK: Sigma, Osterode am Harz, Germany) was employed for protein precipitation from plasma samples.

### 2.2. Materials

Standard substances of SEL and LIN, each with purities exceeding 99%, were procured from LC Laboratories (Woburn, MA, USA). The Zorbax Eclipse Plus C18 HPLC column (150 mm length × 4.6 mm internal diameter, 5 µm particle size) was a product of Agilent (Santa Clara, CA, USA). A guard column, produced by Macherey-Nagel GmbH & Co. (Düren, Germany), was connected before the analytical column. HPLC-grade solvents were acquired from Merck (Darmstadt, Germany), while all other materials were of analytical grade. Human plasma was sourced from the Blood Bank of King Khaled University Hospital in Riyadh, Saudi Arabia, and it was preserved in a freezer at −70 °C until the time of analysis.

### 2.3. Preparation of Standard Solutions and Quality Control Samples

Accurate quantities (10 mg) of SEL and LIN (used as the internal standard: IS) were individually placed into 10 mL calibrated flasks. These materials were dissolved in approximately 8 mL of acetonitrile, and the volumes were adjusted to 10 mL with the same solvent, resulting in stock solutions with a concentration of 1.0 mg mL^−1^. Subsequently, these solutions were diluted with acetonitrile to create working standard solutions of both SEL and LIN with concentrations of 2000 and 200 ng mL^−1^ for SEL and LIN, respectively.

Calibration standard samples were prepared by spiking SEL and LIN (IS) into blank human plasma, yielding final SEL concentrations ranging from 100 to 2000 ng mL^−1^ and a constant IS concentration of 200 ng mL^−1^ across all solutions. The samples were then mixed with an equal volume of methanol, vortexed for 30 s, and centrifuged for 10 min at 13,000 rpm using the Eppendorf Himac Centrifuge (Hamburg, Germany). The resulting supernatants were filtered using syringes equipped with 0.2 µm Millipore filters before analysis. Each sample (10 µL) was injected into the HPLC system for analysis.

Quality control (QC) samples were also prepared at three distinct concentration levels: low, medium, and high, with SEL concentrations set at 100, 400, and 800 ng mL^−1^, respectively. These QC samples were prepared following the same procedures used for the calibration standards. The samples were refrigerated until they were required for analysis, which was conducted on different days, with daily assessment of system suitability parameters.

### 2.4. HPLC Analysis and Statistical Data Treatment

Chromatographic separations utilizing HPLC were achieved utilizing a mobile phase consisting of acetonitrile and acetate buffer solution of pH 5 (50:50, *v*/*v*) delivered to the system in an isocratic mode at a flow rate of 1.0 mL min^−1^. Samples (10 µL aliquots) including SEL standards, blank plasma, blank plasma spiked with LIN as an internal standard (IS), or blank plasma spiked with both SEL and LIN were injected into the HPLC system by the autosampler. The peak area ratio (SEL peak area to LIN peak area) was plotted against the corresponding SEL concentrations to create a calibration curve. This curve, with the peak area ratio on the Y-axis and concentrations on the X-axis, was employed to establish a regression equation for quantifying SEL concentrations in the samples.

The statistical analysis was carried out utilizing Microsoft Excel Software, specifically version 2021 of Microsoft Office 365 (Microsoft Corporation, Redmond, WA, USA). All values were presented as the mean ± standard deviation (SD) or relative standard deviation (RSD). The calibration lines of the HPLC-PDA method underwent regression analysis using the built-in Data Analysis Package within Excel Software, with a significance threshold set at *p* < 0.05. This analysis encompassed the computation of various parameters, including the intercept, slope, correlation coefficient, and variance.

### 2.5. Validation of HPLC-PDA Method

The HPLC-PDA method underwent an extensive validation procedure according to the International Council on Harmonisation (ICH) guidelines for bioanalytical method validation and study sample analysis, M10 [8]. This validation process was conducted to assess the method’s validity in terms of linearity, sensitivity, precision, accuracy, selectivity, and ruggedness for quantifying SEL in plasma samples.

#### 2.5.1. Assessment of Linearity and Sensitivity

For linearity evaluation, three separate calibration curves were independently constructed to derive regression equations and their corresponding determination coefficients. The weighting factor of 1/x^2^ was applied during the linear regression analysis for the calibration curve. These coefficients’ values were employed as indicators to evaluate the method’s linearity. The method’s sensitivity was determined by calculating the limit of detection (LOD) and limit of quantification (LOQ) using the following formula: LOD or LOQ = ×SDa/b, where × is 3.3 for LOD and 10 for LOQ. In this context, SDa refers to the standard deviation of the calibration line intercept, while b represents its slope.

#### 2.5.2. Determination of Precision and Accuracy

Intra-assay precision and accuracy were assessed by analyzing six replicate samples of each QC sample at three distinct levels (low, medium, and high) within a single batch. Similarly, inter-assay precision and accuracy were determined by analyzing three replicate samples of each QC sample at each level over three consecutive days. Precision was evaluated using the percentage of the relative standard deviation (RSD, %) obtained from the replicate measurements. Accuracy was expressed as the percentage of recovery values (recovery, %), calculated by comparing the measured concentrations to the designated concentrations.

#### 2.5.3. Assessment of Method Ruggedness

To evaluate the robustness of the method, the analysis of SEL was carried out under the specified operating conditions by two different analysts over three consecutive days. The results obtained were presented in terms of the RSD %, offering an indication of the variability observed in the data.

#### 2.5.4. Stability Studies of SEL in Samples

The stability of SEL was assessed under various conditions and environments (autosampler, bench-top, freeze–thaw, short-term storage, and long-term storage). Autosampler stability was investigated by storing QC samples under autosampler conditions for approximately 48 h before analysis. Bench-top stability was evaluated by leaving plasma samples at room temperature for around 6 h. Freeze–thaw stability was determined by subjecting samples to three cycles of freezing (at −70 °C) and thawing (at room temperature).

For short-term and long-term stability assessments, samples were stored at 8 °C (for 30 days) and −70 °C (for 60 days), respectively, before being analyzed using the proposed HPLC-PDA method. The stability of stock solutions and working solutions of SEL was tested by storing them at room temperature for 24 h and at refrigerator temperature (below 10 °C) for 30 days prior to analysis. Freshly prepared calibration standards were utilized for all stability tests. Samples were considered stable in plasma if the deviation from the mean calculated concentration of stability QC was within ±15%.

### 2.6. Pharmacokinetic Study in Rats

Five healthy male Wistar rats, with an average weight of 250 ± 30 g, were housed under standard conditions at a room temperature of 25 °C and an average relative humidity of 50%. The rats were orally administered SEL at a dose of 25 mg/kg dissolved in a solution of 1% dimethyl sulfoxide/saline. Blood samples of approximately 300 μL each were collected before dosing and at specified time intervals following drug administration (ranging from 0.25 to 12 h) into heparinized tubes. Plasma was obtained by centrifuging the blood samples at 4500 rpm and 4 °C for 15 min, then stored at −20 °C until analysis.

The animal study adhered strictly to the guidelines of the Research Ethics Committee (RCE) for experiments involving live animals at King Saud University (Riyadh, Saudi Arabia), under reference No: KSU-SE-20-51. Pharmacokinetic parameters were determined using the non-compartmental model with the PKSolver tool, an Add-In for Microsoft Excel (version 2018, part of Microsoft Office 365 by Microsoft Corporation (Redmond, WA, USA). The parameters calculated included maximum plasma concentration (C_max_), time to reach C_max_ (T_max_), volume of distribution (V_d_), elimination rate constant (K_el_), elimination half-life (t_1/2_), clearance (CL), area under the curve from 0 to 24 h and from 0 to infinity (AUC_0–24_; AUC_0-∞_), and mean residence time (MRT).

### 2.7. Assessment of Environmental Sustainability of HPLC-PDA Procedures

The environmental sustainability (eco-friendliness/greenness) of HPLC-PDA procedures was evaluated using three different assessment tools: the Analytical Eco-Scale (AES) [9], the Green Analytical Procedure Index (GAPI) [10], and Analytical Greenness (AGREE) [11].

The AES tool assesses the environmental impact of analytical processes based on four intrinsic parameters. Each parameter incurs penalty points (PPs) reflecting its adverse environmental effects, with guidelines provided for calculating these points. By deducting the total PPs from 100, the tool indicates the eco-friendliness of the procedure. The AES categorizes procedures into green (total score > 75 points), reasonably green (total score of 50–75 points), and inadequately green (total score < 50 points) based on the resulting score.

The GAPI tool evaluates the entire analytical process across 15 parameters (1–15), spanning activities from sample collection to waste generation/treatment post-analysis. A pictogram with 15 segments visually represents these parameters, with each segment assigned a color (green, yellow, or red) to signify its environmental impact. Green denotes sustainability, while red indicates a lack of environmental friendliness; detailed guidelines for the assignment of the colors are provided.

The AGREE tool (v 0.5 beta) is user-friendly software that assesses the environmental sustainability of analytical methods based on the 12 principles of Green Analytical Chemistry (GAC). The results are automatically presented in a circular pictogram format, with each segment color-coded from deep green (=1) to deep red (=0) based on its impact on assay sustainability. The total score displayed at the pictogram’s center is computed as a fraction of unity.

## 3. Results and Discussion

### 3.1. Strategy for Method Development

Following the introduction of SEL and its notable efficacy and benefits in treating different types of cancers, researchers worldwide have shown interest in refining the findings to improve therapeutic outcomes, reduce side effects, enhance target specificity, overcome resistance, explore combination therapies, and advance personalized medicine approaches in the treatment of various diseases. To achieve these objectives, an appropriate analytical method for quantifying SEL in plasma is essential. The availability of such a method would be required for several reasons. (1) Pharmacokinetic studies: Understanding the pharmacokinetics of SEL, including its absorption, distribution, metabolism, and excretion in the body requires accurate quantification of the drug in plasma. These data are vital for determining how the drug behaves in the body over time and under different conditions. (2) Therapeutic monitoring: Monitoring the concentration of SEL in plasma is crucial for ensuring that patients can receive the correct dose for effective treatment. By measuring plasma levels, healthcare providers can optimize dosing regimens to maximize therapeutic benefits while minimizing potential side effects. (3) Safety and efficacy: Quantifying SEL levels in plasma helps in assessing the drug’s safety and efficacy profile. By measuring drug concentrations in plasma over time, researchers can assess key pharmacokinetic parameters such as absorption, distribution, metabolism, and excretion. These data aid in determining the drug’s bioavailability, half-life, and clearance rates, which are essential for optimizing dosage regimens and predicting potential drug interactions. Monitoring SEL levels in plasma also enables researchers to correlate drug exposure with therapeutic effects and potential adverse reactions, providing valuable insights into the drug’s overall safety and efficacy in a clinical setting. (4) Compliance and regulatory requirements: Regulatory agencies often require detailed pharmacokinetic data during the drug approval process. An analytical method for quantifying SEL in plasma is necessary to meet these regulatory standards and demonstrate the drug’s safety and efficacy.

Given the importance of assessing plasma concentrations of SEL, and the absence of bioanalytical methods for SEL quantification in plasm, this study aimed to address this gap and develop a new method for quantification of SEL in plasma. A previous study [5] demonstrated that SEL molecules have distinct UV light absorption capabilities; thus, this study was designed to utilize this property in developing a sensitive PDA-assisted HPLC method for quantifying SEL in plasma samples. Additionally, building on our laboratory’s success in developing simple non-extractive procedures for HPLC analysis of pharmaceuticals in plasma samples, this study aimed to employ the same approach in the development of the HPLC-PDA method for SEL. Furthermore, the study was intended and designed to develop a sustainable HPLC method by adopting two main approaches that minimize negative environmental impact. The first approach was the selection of environmentally green solvents with lower toxicity, like mobile phase with a highly water-based composition. The second approach was reducing solvent consumption by developing a method with a short run time while maintaining the best possible chromatographic separations.

### 3.2. Optimization of the HPLC-PDA Conditions

#### 3.2.1. Selection of Detection Wavelength and Internal Standard

The UV-absorption spectrum of SEL demonstrated that SEL exhibits strong absorption characteristics within the range of 200–325 nm, showing a maximum absorption peak at 230 nm (Figure 1B). Therefore, this native UV absorption at 230 nm was explored for detection by the PDA detector in this study. A study in our laboratory highlighted the UV absorption characteristics of 12 molecules of the tyrosine kinase inhibitors family, demonstrating that LIN has a significant UV absorption capability overlapping with that of SEL. The chemical structure of LIN and its UV absorption spectrum are shown in Figure 1A,B, respectively. Due to this correspondence, LIN was selected as the internal standard (IS) for inclusion in the HPLC-PDA method detailed in this study.

#### 3.2.2. Mobile Phase Composition

Experiments were conducted with the aim of establishing the most appropriate mobile phase composition and flow rate. Preliminary separation experiments were conducted utilizing an analytical HPLC Zorbax Eclipse Plus C18-column (250 mm length × 3.9 mm i.d., 5 μm particle diameter) maintained at 25 ± 2 °C, and an isocratic mode for elution of a mobile phase. The initial mobile phase consisted of a mixture of methanol and acetate buffer at pH 5 (80:20, *v*/*v*) with a flow rate of 1 mL min^−1^. Under these conditions, SEL eluted relatively quickly at 4.22 min; however, its peak overlapped with that of IS, which eluted at 4.31 min. Subsequently, the methanol content in the mobile phase was adjusted to 70%; however, this alteration resulted in excessive elongation of the retention time of SEL, as it eluted at 8.05 min and the run time was ~15 min. Further refinements were made by replacing methanol with acetonitrile while maintaining the acetate buffer content at 70%. This adjustment facilitated a distinct separation between SEL and IS within 7.5 min, with SEL eluting at 3.52 min. Given the necessity of elongating the retention time of SEL to prevent any potential interference with early-eluting plasma components in the intended HPLC-PDA method, the acetonitrile content was reduced from 70% to 50%. This change resulted in a more favorable run time (10 min), excellent separation, and reasonable retention times of 5.97 and 8.23 min for SEL and IS, respectively. This mobile phase, composed of acetonitrile–ammonium acetate, pH 5 (50:50, *v*/*v*), was used for the subsequent experiments.

#### 3.2.3. Column Packing and Dimensions

**Figure 2:**
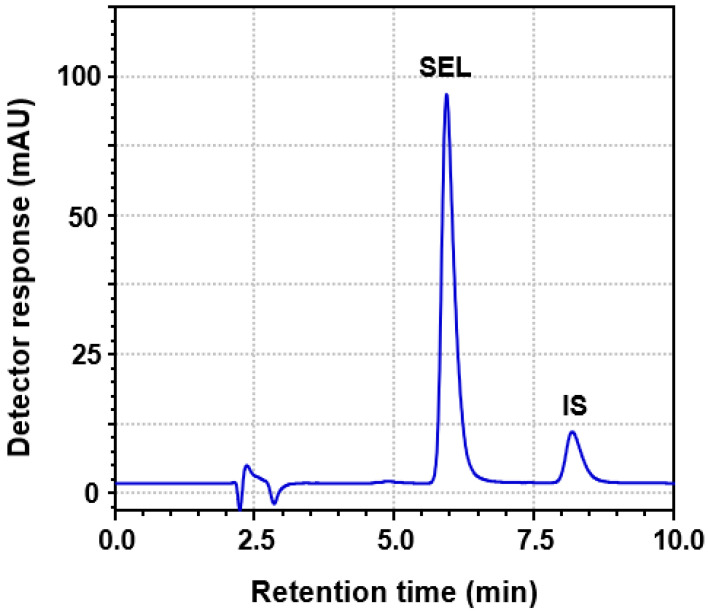
A representative chromatogram of standard solution containing SEL and IS. The concentrations of SEL and IS were 500 and 100 ng mL^−1^, respectively. mAU is the detector response in millivolts as arbitrary units.

To select the most appropriate column packing and dimensions, other reversed-phase columns including phenyl columns (250 mm length × 4.6 mm i.d., 5 μm particle diameter; manufactured by Phenomenex Inc. (Torrance, CA, USA) and Nucleosil CN (250 mm length × 4.6 mm i.d., 5 µm particle diameter) manufactured by Macherey-Nagel GmbH & Co. (Düren, Germany) were tested. The results demonstrated that Zorbax Eclipse Plus C18-column (250 mm length × 3.9 mm i.d., 5 μm particle diameter) was the best column, and thus, it was selected for all the subsequent work. Under these conditions, a good resolution between SEL and IS was achieved within a reasonably short run time (10 min); SEL and IS were eluted as sharp, narrow, symmetric peaks at 5.97 and 8.23 min, respectively (Figure 2). The system suitability parameters (capacity factor, separation factor, resolution factor, peak symmetry factor, and the number of theoretical plates) were measured, and the results are given in Table 1.

### 3.3. Results of HPLC-PDA Method Validation 

Following the ICH guidelines for bioanalytical method validation [8], validation of the proposed HPLC-PDA was carried out across all validation parameters. These parameters were linearity, sensitivity (LOD, LOQ), accuracy, precision, stability, selectivity, carryover, and ruggedness.

#### 3.3.1. Linearity

**Figure 3:**
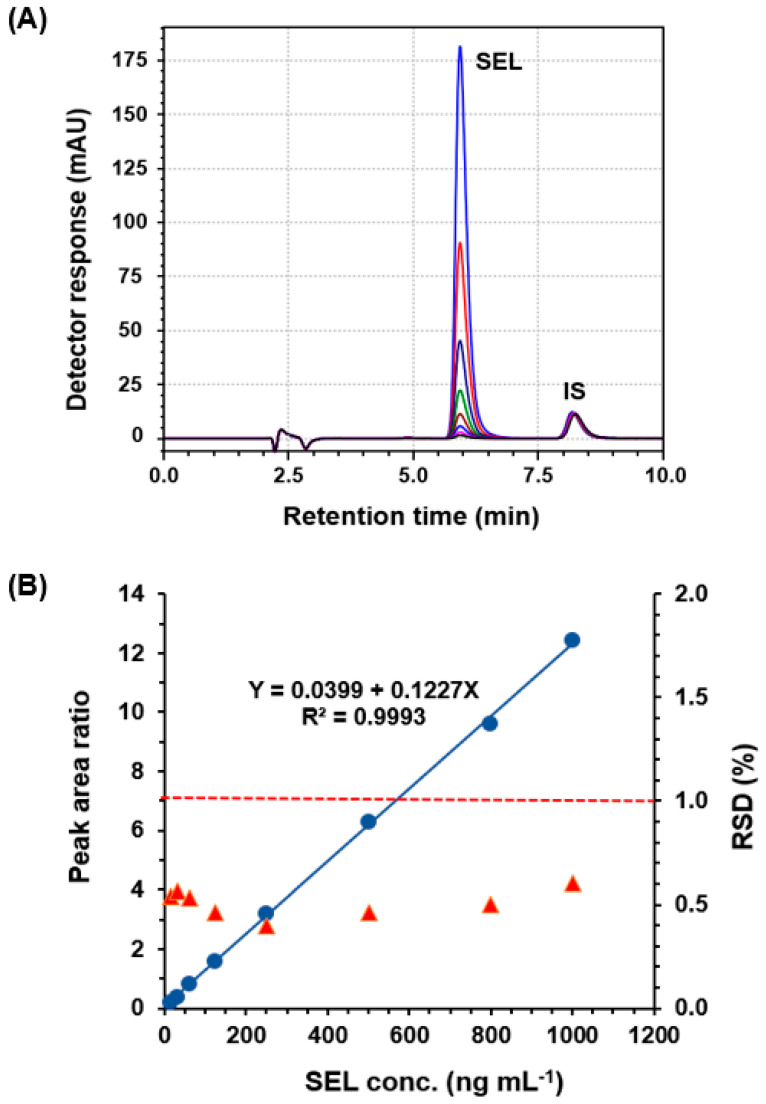
Panel (**A**): Overlaid chromatograms of standard solutions containing varying concentrations (50–1000 ng mL^−1^) of SEL and a fixed concentration of IS (100 ng mL^−1^). Panel (**B**): the calibration curve (Blue circles, ●; on the left axis) and precision profile, expressed as RSD, % (red triangles, ▲; on the right axis) of the HPLC-PDA method for the determination of SEL.

Under the specified conditions, a range of concentrations of SEL was tested. The chromatograms overlaying standard solutions with varying concentrations are displayed in Figure 3A. To generate the calibration curve, the peak area ratios of SEL to that of the IS were plotted against the corresponding SEL concentrations, as illustrated in Figure 3B. Linear regression analysis was applied to the data, revealing strong linear correlations with a high determination coefficient (r^2^) and minimal intercept value. These outcomes indicate that the method established exhibits significant linearity. The linear equation is as follows:

Y = 0.0399 + 0.1227X (r^2^ = 0.9993)

where Y represents the peak area ratio and X denotes the SEL concentration. A summary of the linearity and statistical parameters for each analyte is provided in Table 2.

#### 3.3.2. Sensitivity

According to the method’s criteria, the limits of detection (LOD) and quantification (LOQ) were computed, and the results are detailed in Table 2. The calculated LOD and LOQ values were 21.8 and 66.1 ng mL^−1^, respectively. These results affirm the method’s sensitivity in accurately measuring therapeutic concentrations of SEL. This deduction was drawn from the observed plasma levels of SEL in patients, which ranged between 307 and 3783 ng mL^−1^, after oral administration of 800 mg dose [12].

#### 3.3.3. Accuracy and Precision

Table 3 summarizes the accuracy (expressed as recovery %) and precision (expressed as RSD %) results for three QC sample concentrations (100, 400, and 800 ng mL^−1^). Intra-day recovery values ranged from 98.26% to 103.25%, while inter-day recovery values varied from 96.44% to 102.61%. In terms of precision, intra-day RSD values ranged from 1.24% to 2.58%, with inter-day RSD values falling between 1.36% and 5.37%. These findings indicate that the proposed HPLC-PDA method complies with the accuracy and precision standards outlined in the ICH guidelines for bioanalytical method validation.

#### 3.3.4. Ruggedness

Ruggedness was evaluated concerning analyst-to-analyst and day-to-day variation. The results, presented in Table 4, exhibited recovery values spanning from 96.8% to 103.1%, with RSD values not exceeding 3.2%. This underscores the ruggedness and reproducibility of the proposed method.

#### 3.3.5. Selectivity

**Figure 4:**
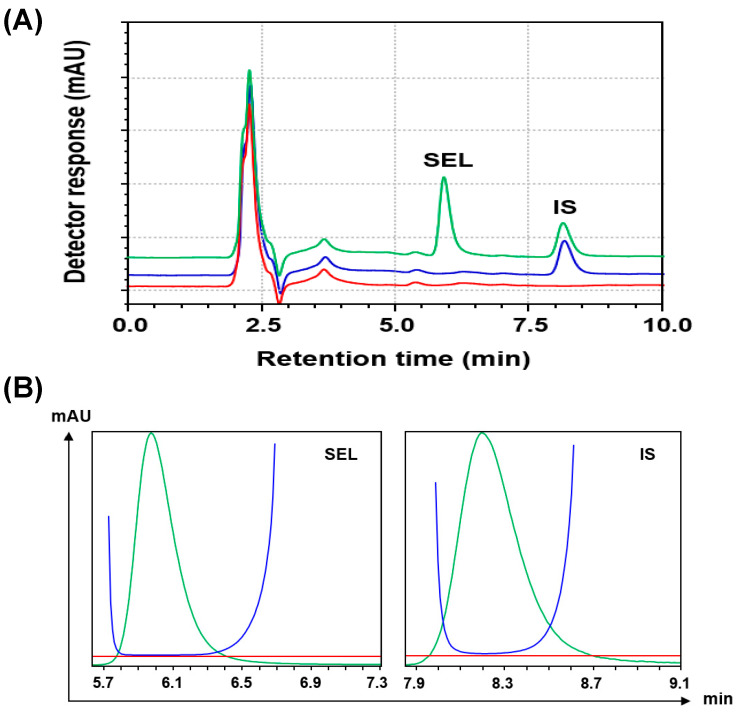
Panel (**A**): Representative chromatograms of blank (drug-free) human plasma (red line), plasma spiked with IS at a concentration of 100 ng mL^−1^ (blue line), plasma spiked with SEL and IS at concentrations of 250 and 100 ng mL^−1^, respectively (green line). Panel (**B**): The purity plots of the SEL and IS peaks. The red, green, and blue curves are the threshold (base) lines, peaks, and purity curves, respectively.

The method’s selectivity was evaluated by examining blank SEL-free human plasma samples. In Figure 4, chromatograms representing three distinct conditions were displayed: a SEL-free sample (without SEL, without IS), a sample from blank plasma spiked with IS at a concentration of 100 ng mL^−1^, and a sample from blank plasma spiked with SEL at a concentration of 250 ng mL^−1^ and IS at 100 ng mL^−1^. The chromatograms in Figure 4 demonstrated the absence of any interfering peaks at the expected retention times for both SEL and IS. Additionally, the purity plots of the peaks were generated (Figure 4B), and the corresponding numerical values were computed and are presented in Table 1. In the peak purity plots (Figure 4B), the purity curves do not rise above the threshold lines. This suggests that any spectral variances observed are solely due to background noise, indicating the presence of a singular compound within the chromatographic peak. The peak purity indices and single point thresholds for all peaks were close to 1, affirming the purity of the peaks and the absence of any co-eluted compounds alongside the targeted analytes (SEL and IS).

#### 3.3.6. Stability Studies of SEL in Plasma Samples

The stability investigations conducted on SEL (as shown in Table 5) demonstrated that SEL within spiked human plasma samples remained stable for at least 6 h at room temperature and 48 h when stored under autosampler conditions. Furthermore, SEL exhibited stability across three freeze–thaw cycles and when stored in a refrigerator at 8 °C for 30 days, and in a freezer at −70 °C for 60 days. These findings are supported by the recovery values falling within the range of 96.58–104.16% and the RSD values not surpassing 6.12%.

### 3.4. Results of Pharmacokinetic Study in Rats

**Figure 5:**
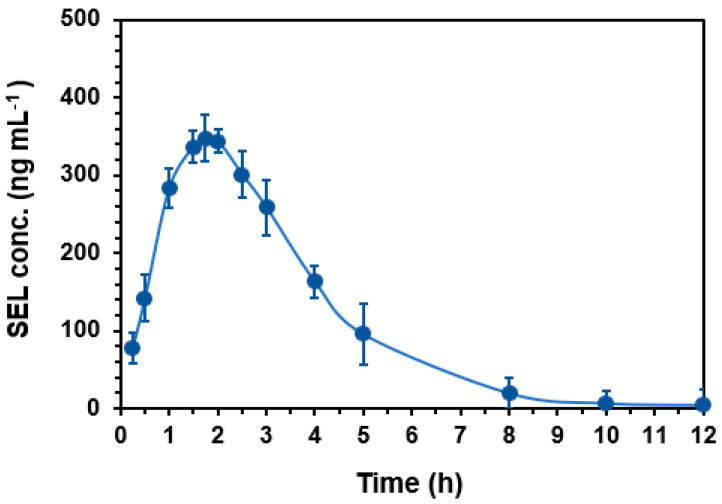
Concentration-time profile of SEL in rats after single oral gavage administration at a dose of 25 mg kg^−1^. Concentrations are the means of 5 rats ± SD.

The proposed HPLC-PDA method was employed for the quantification of SEL in plasma samples obtained from rats after their oral administration with SEL at a dose of 25 mg/kg. Rats were selected in this study because they were proved to be the most appropriate animal model for studying human biology due to their high degree of genomic and physiologic similarities to humans [13]. The mean plasma concentration (in ng mL^−1^) versus time (h) profile of SEL in rats is shown in Figure 5, and the main pharmacokinetic parameters explored are summarized in Table 6.

After oral administration of SEL in rats, it was rapidly absorbed and reached a maximum plasma concentration (C_max_) of 347.41 ng mL^−1^ after 1.75 h (t_max_) and had an eliminated half-life (t½) of 1.49 h. The determined volume of distribution (V_d_) of SEL was low (0.04 L/kg), indicating that SEL is primarily confined to the bloodstream and has limited distribution into tissues. The clearance (CL) of SEL was found to be low at 0.02 L/h/kg, indicating that SEL is eliminated from the body mainly via the renal elimination route and at a relatively slow rate. The ratios of AUC (AUC_0–t_/AUC_0–∞_) were found to be 99.12%, demonstrating that the present HPLC-PDA method was sufficiently sensitive to cover the elimination phase of SEL. The bioavailability (F) of SEL was calculated based on AUC and expressed as the percentage ration of AUC found after oral administration to the AUC found after intravenous administration of the same dose. The F value was found to be 86.5%, indicating the high bioavailability of SEL. These findings reveal the validity of the HPLC-PDA for assessing the pharmacokinetics of SEL. Additionally, the acquired pharmacokinetic values offer important information for refining and optimizing SEL therapy, assisting with the dosing strategy, and promoting safe and effective treatment.

### 3.5. Eco-Friendliness/Greenness of HPLC-PDA Procedures

Within GAC, a strong emphasis is placed on employing environmentally friendly analytical techniques or procedures throughout the entire analytical process, spanning from sample preparation to the final determination of samples [14,15]. This involves decreasing the utilization of harmful chemicals, reducing waste production, and preserving energy. While developing the proposed HPLC-PDA method, efforts were made to reduce the organic solvent content in the mobile phase and minimize the run time to lower solvent consumption and waste generation, all while upholding the optimal resolution between the SEL and IS. In this study, the sustainability of the proposed HPLC-PDA method in terms of its eco-friendliness was evaluated to offer a detailed and thorough assessment of the environmental impact of analytical procedures. To assess the greenness of the proposed method, three distinct metric tools, AES [9], GAPI [10], and AGREE [11], were utilized. The subsequent section delves into the outcomes derived from these tools.

#### 3.5.1. Analytical Eco-Scale

The results from the AES tool are detailed in Table 7, where penalty points (PPs) were allotted for the organic solvent (acetonitrile) and reagent (ammonium acetate) at 2 and 4 PPs respectively, based on their quantities. Regarding their hazard impact, acetonitrile and ammonium acetate were assigned 1 and 2 PPs, respectively. Energy consumption by HPLC-PDA was given 1 PP, while the occupational hazards parameters were not given any PP for compliance with the tool’s criteria. Waste production and treatment parameters accumulated subtotals of four and three PPs, respectively, as the proposed HPLC-PDA method yields ≤ 10 mL of untreated waste per sample. Consequently, the HPLC-PDA procedures accrued a total of 17 PPs, translating to an Eco-Scale score of 83 (100 − 17). This significant rating underscores the commendable environmental sustainability of the HPLC-PDA procedures, aligning well with the AES tool’s standards.

#### 3.5.2. Green Analytical Procedure Index

**Figure 6:**
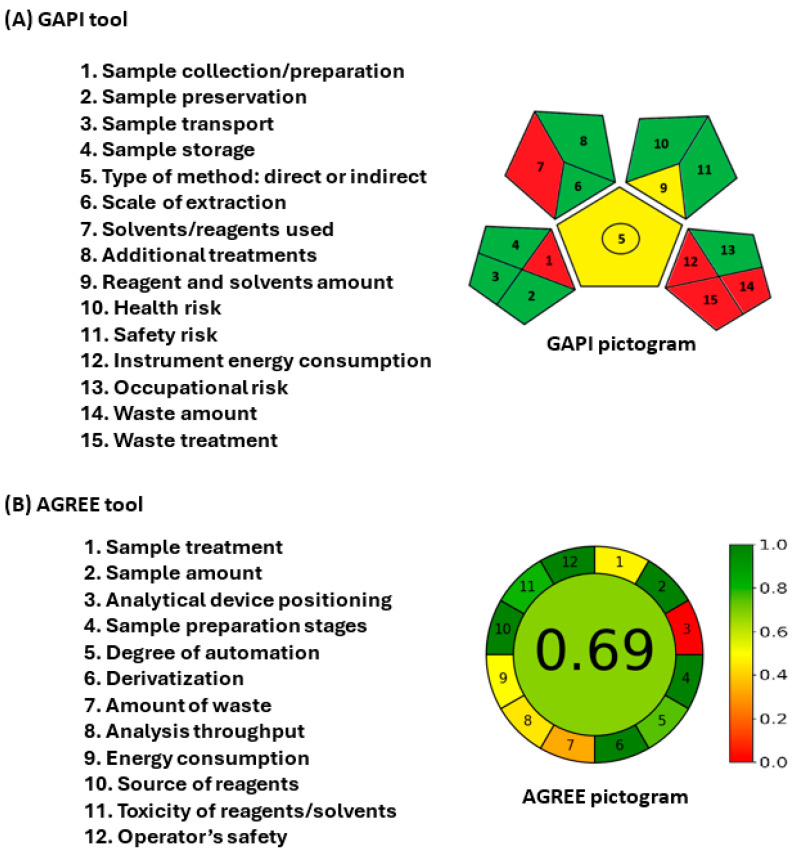
The evaluation of the greenness of the proposed HPLC-PDA for the determination of SEL by GAPI (**A**) and AGREE (**B**) tools. The evaluation parameters and pictograms are given in the left-hand and right-hand sections of each panel.

The GAPI tool results are visually represented in a pictogram comprising 15 assessment parameters (Figure 6, upper section). Within these parameters, five (1, 7, 12, 14, and 15) are highlighted in red on the pictogram, indicating their non-compliance with the GAC criteria. Parameter 5 is displayed in yellow because the method is a direct approach for quantitative purposes. Parameters 6 and 10 are also displayed in yellow as they involve microscale sample extraction and solvent use posing a moderate health risk. The remaining parameters are depicted in green, showcasing their full adherence to the environmentally friendly procedure requirements outlined in the GAPI tool guidelines. In summation, 8 out of 15 parameters (53.3%) are green, 2 parameters (13.3%) are yellow, and 5 parameters (33.3%) are red. These findings underscore the satisfactory eco-friendliness of the procedures within the proposed HPLC-PDA method.

#### 3.5.3. Analytical Greenness

The pictogram derived from the AGREE tool is illustrated in Figure 6 (lower section). Parameter 1, which is related to sample treatment, is displayed in yellow due to the manual handling of samples. Parameter 3, addressing device positioning (online or offline), is highlighted in red because the analysis of the HPLC system was conducted offline. Parameter 7 is portrayed in a reddish-yellow hue as the procedures generated 10 mL of waste mobile phase. Parameter 8 appears as a dark yellow shade because the run time was 10 min. Parameter 9 is depicted in yellow based on the energy consumption of the HPLC system. The remaining parameters are depicted in green, indicating conformity with environmentally friendly practices. The total score attained was 0.69 out of 1, showcasing a high level of eco-friendliness for the proposed HPLC-PDA method.

## 4. Conclusions

This study introduces the first method for quantifying SEL in human plasma using the HPLC-PDA system. The proposed HPLC-PDA exhibits sufficient sensitivity to accurately and precisely measure SEL in plasma at concentrations as low as 66.1 ng mL^−1^. The methodology avoided any labor-intensive liquid–liquid or solid-phase extractions, opting instead for straightforward simple pretreatment steps involving protein precipitation from plasma samples using methanol. Chromatographic separation of SEL and IS was accomplished through a simple isocratic elution within a relatively brief run time of approximately 10 min. SEL and IS were detected at retention times of 5.97 and 8.23 min, respectively. Extraction recovery percentages ranged from 96.44% to 104.38%. Notably, the method exhibited high precision, with RSD values remaining at ≤5.37%. These characteristics support efficient and reliable high-throughput analysis, streamlining sample processing across various clinical laboratories. Moreover, the proposed HPLC-PDA method was evaluated using three green metric tools: ESA, GAPI, and AGREE, yielding scores of 83/100, 8/15, and 0.69/1, respectively. These outcomes underscore the minimal environmental impact of the system while upholding analytical performance standards. The HPLC-PDA system holds the potential for refining pharmacokinetics, therapeutic drug monitoring, and laying a foundation for future investigations into drug–drug interactions in human subjects.

## Figures and Tables

**Table 1:** The system suitability parameters and peak purity profiling of the HPLC-PDA method for the determination of SEL.

Parameter	Value	
	SEL	IS
System suitability		
Retention time of SEL (min)	5.97	8.23
Capacity factor (*K*’)	1.75	2.75
Separation factor (α)	1.71	1.61
Resolution factor (Rs)	5.10	4.58
Peak asymmetry factor	1.64	1.51
Number of theoretical plates/m	11,925	14,485
Peak purity		
Impurity detection	Not detected	Not detected
Purity index	1.000000	0.999997
Single point threshold	0.999889	0.990984
Minimum purity index	112	8214

**Table 2:** Calibration parameters for the determination of SEL by the proposed HPLC-PDA method.

Parameter	Value
Linear range (ng mL^−1^)	50–1000
Intercept (a)	0.0399
Standard deviation of intercept (SD_a_)	0.8112
Slope (b)	0.1227
Standard deviation of slope (SD_b_)	0.0124
Determination coefficient (r^2^)	0.9993
Limit of detection (LOD, ng mL^−1^)	21.8
Limit of quantitation (LOQ, ng mL^−1^)	66.1

**Table 3:** Intra-assay and inter-assay precision and accuracy for determination of SEL in spiked human plasma.

Nominal SEL Conc. (ng mL^−1^)	Intra-Day ^a^		Inter-Day ^a^	
	Accuracy (Recovery, %)	Precision (RSD%)	Accuracy (Recovery, %)	Precision (RSD%)
100	103.25	2.54	102.61	4.51
400	99.52	1.24	104.38	1.36
800	98.26	2.58	96.44	5.37

^a^ Values are the means of 6 replicates.

**Table 4:** Ruggedness of the proposed HPLC-PDA for the determination of SEL.

Parameters	Recovery (% ± RSD) ^a^
Analyst-to-analyst	
Analyst-1	97.6 ± 1.5
Analyst-2	101.2 ± 2.2
Day-to-day	
Day-1	102.5 ± 1.8
Day-2	103.1 ± 1.6
Day-3	96.8 ± 3.2

^a^ Values are the means of three determinations.

**Table 5:** Stability data of SEL in human plasma.

Stability	Recovery (%) ^a^	Precision (RSD, %) ^a^
Bench top (6 h)	98.14	6.12
Autosampler (48 h)	100.16	5.22
Freeze–thaw (3 cycle)	96.58	4.52
30 days at 8 °C	101.52	2.81
60 days at −80 °C	104.16	3.26

^a^ Values are the means of 3 determinations.

**Table 6:** The pharmacokinetic parameters of SEL in rat plasma after oral administration of 25 mg/kg.

Parameter	Unit	Value ^a^
Maximum plasma concentration (C_max_)	ng/mL	347.41
Time required for maximum plasma concentration (T_max_)	h	1.75
Volume of distribution (V_d_)	L/kg	0.04
Elimination rate constant (K_el_)	1/h	0.46
Elimination half-life time (t_1/2_)	1/h	1.49
Clearance (CL)	L/h/kg	0.02
Area under curve from time 0 to last concentration (AUC_0–t_)	ng·h/mL	1315.25
Area under curve at infinite time (AUC_0-∞_)	ng·h/mL	1326.02
Area under curve ration (AUC_0–t_/AUC_0–∞_)	%	99.12
Mean residence time (MRT)	h	3.09
Bioavailability (F)	(%)	86.5

^a^ Values are the means of five determinations ± SD.

**Table 7:** Analytical Eco-Scale for assessing the greenness of the proposed HPLC-PDA method for the simultaneous determination of SEL.

Eco-Scale Score Parameters	Penalty Points (PPs)
Solvent/reagent	
Amount: acetonitrile (≤10 mL per sample)	2
Ammonium acetate buffer (≤10 mL per sample)	4
Hazards: acetonitrile	1
Ammonium acetate buffer	2
Instrument: Energy used (kWh per sample)	
HPLC-PDA	1
Occupational hazards	
Analytical process hermetic	0
Emission of vapors and gasses to the air	0
Waste	
Production (≤10 mL per sample)	4
Treatment (no treatment involved)	3
Total PPs	17
Eco-Scale score	83

## Data Availability

All data are available from the corresponding author (idarwish@ksu.edu.sa).
